# Effects of high doses of selenium, as sodium selenite, in septic shock: a placebo-controlled, randomized, double-blind, phase II study

**DOI:** 10.1186/cc5960

**Published:** 2007-07-06

**Authors:** Xavier Forceville, Bruno Laviolle, Djillali Annane, Dominique Vitoux, Gérard Bleichner, Jean-Michel Korach, Emmanuel Cantais, Hugues Georges, Jean-Louis Soubirou, Alain Combes, Eric Bellissant

**Affiliations:** 1 Service de Réanimation Polyvalente, Centre Hospitalier de Meaux, Hôpital Saint Faron, 6–8 rue Saint Fiacre, 77104 Meaux, France; 2 Centre d'Investigation Clinique INSERM 0203, Unité de Pharmacologie Clinique, Hôpital de Pontchaillou, CHU de Rennes et Université de Rennes 1, 2 rue Henri le Guilloux, 35033 Rennes, France; 3 Service de Réanimation Médicale, Hôpital Raymond Poincaré, 104 boulevard Raymond Poincaré, 92380 Garches, France; 4 Service de Biochimie A, Hôpital Saint-Louis, avenue Claude Vellefaux, 75475 Paris cedex 10, France; 5 Service de Réanimation Polyvalente, Centre Hospitalier Victor Dupouy, 69 rue du Lieut-Col Prudhon, 95107 Argenteuil cedex, France; 6 Service de Réanimation Polyvalente, Centre Hospitalier, 51 rue du Commandant Derrien, 51005 Châlons en Champagne cedex, France; 7 Hôpital d'Instruction des Armées Sainte Anne, boulevard Sainte Anne, 83800 Toulon Naval, France; 8 Centre Hospitalier Gustave Dron, 135 rue du Président Coty, 59200 Tourcoing, France; 9 Hôpital d'Instruction des Armées Desgenettes, 108 boulevard Pinel, 69003 Lyon, France

## Abstract

**Introduction:**

Sepsis is associated with the generation of oxygen free radicals and (lacking) decreased selenium plasma concentrations. High doses of sodium selenite might reduce inflammation by a direct pro-oxidative effect and may increase antioxidant cell capacities by selenium incorporation into selenoenzymes. We investigated the effects of a continuous administration of high doses of selenium in septic shock patients.

**Methods:**

A prospective, multicentre, placebo-controlled, randomized, double-blind study was performed with an intention-to-treat analysis in severe septic shock patients with documented infection. Patients received, for 10 days, selenium as sodium selenite (4,000 μg on the first day, 1,000 μg/day on the nine following days) or matching placebo using continuous intravenous infusion. The primary endpoint was the time to vasopressor therapy withdrawal. The duration of mechanical ventilation, the mortality rates in the intensive care unit, at hospital discharge, and at 7, 14, 28 and 180 days and 1 year after randomization, and adverse events were recorded.

**Results:**

Sixty patients were included (placebo, *n *= 29; selenium, *n *= 31). The median time to vasopressor therapy withdrawal was 7 days in both groups (95% confidence interval = 5–8 and 6–9 in the placebo and selenium groups, respectively; log-rank, *P *= 0.713). The median duration of mechanical ventilation was 14 days and 19 days in the placebo and selenium groups, respectively (*P *= 0.762). Mortality rates did not significantly differ between groups at any time point. Rates of adverse events were similar in the two groups.

**Conclusion:**

Continuous infusion of selenium as sodium selenite (4,000 μg on the first day, 1,000 μg/day on the nine following days) had no obvious toxicity but did not improve the clinical outcome in septic shock patients. Trial Registration = NCT00207844.

## Introduction

Septic shock – an uncontrolled systemic host response to invasive infection leading to multiple organ failure – is a public health issue because of its frequency, cost and 45% mortality rate [1,2]. The physiopathology of septic shock is better understood with increasing data supporting the key role of oxidant stress, especially on endothelium damage [3-5]. In severe sepsis patients or in systemic inflammatory response syndrome patients, there is an early 40% decrease in plasma selenium concentrations that could be associated with a decrease of antioxidant defences [6]. Recent data suggest that selenium administration as sodium selenite could induce a dose-dependent favourable effect on the clinical outcome and survival in septic shock, especially in severe septic shock patients [3,7-10].

Selenium can induce two fundamental types of effects: antioxidant, through its incorporation into selenoenzymes; and pro-oxidant, through the direct effects of selenocompounds.

Selenoenzymes, which require one atom of selenium at their active site to be functional, protect cells against damages related to oxidative stress [11,12]. Among them, selenoprotein P may be involved in endothelium protection during sepsis [13]. These ubiquitous enzymes regulate many intracellular metabolic pathways such as arachidonic acid cascade, NF-κB transcription activation, transcriptional activities and mitochondrial functions [14-17]. Owing to their numerous biological functions, a severe selenium deficiency may be lethal [18].

In contrast, selenocompounds, especially sodium selenite, can display pro-oxidant properties that may be toxic [19,20]. Indeed, selenium was initially known as a toxic element in animal poisoning by selenium-rich plants [21]. In animals, the minimum lethal dose for intravenous administration of sodium selenite is between 1.5 and 3 mg/kg [22,23]. In humans, acute lethal poisonings are rare [22-25], with observed toxic effects clinically similar to those of arsenic [25]. The minimum lethal dose seems to be similar to that for animals [22]. The toxicity of selenium compounds, especially sodium selenite, is considered to be related to its pro-oxidant properties [19,20,26,27]. The daily nutritional intakes to avoid deleterious effects have been established as 400 μg for the tolerable-upper-intake level and as 800 μg for the no-adverse-event level [28], whereas a unique ingestion of 4 mg selenium is considered nontoxic in a healthy man [22]. In the case of oxidative stress related to septic shock, administration of more than 700 μg/day selenium is currently not recommended due to the pro-oxidative effect of selonocompounds [23,29-31]. In clinical trials, however, daily doses up to 1,000 μg have been repeatedly used without detectable adverse effects [8,10].

In septic shock treatment, the pro-oxidant properties of selenite may be interesting as they may temporarily reduce excessive inflammation by inhibiting NF-κB to DNA binding [32,33] or by inducing a proapoptotic effect on activated circulating cells [13,20,34]. We therefore designed the present study to assess the efficacy and safety of a continuous infusion of sodium selenite initially given at a pro-oxidative high dose, cautiously (lacking) administered continuously, followed by an antioxidative lower dose in septic shock patients.

## Methods

### Study design

A prospective, placebo-controlled, randomized, double blind, phase II study was conducted in seven centres in France. The protocol was approved by the Consultative Committee for the Protection of People in Biomedical Research (Comité Consultatif de Protection des Personnes dans la Recherche Biomédicale) of Saint-Germain en Laye, France on 15 March 2001.

### Patients

Patients older than 18 years and hospitalized in participating intensive care units (ICUs) were enrolled in the study if they met the following criteria: severe documented infection, as evidenced by one or more of a positive culture or Gram stain of a normally sterile body fluid, of a clinical patent focus of infection (for example, faecal peritonitis, community pneumonia) and of a nosocomial documented infection (for example, ventilation-acquired nosocomial pneumonia or catheter-related infection); a need for mechanical ventilation; severe septic shock, defined as circulatory failure that required at least 1,000 ml fluid replacement in the previous 24 hours and was treated for at least 1 hour with more than 15 μg/kg/min dopamine or more than 0.2 μg/kg/min epinephrine or norepinephrine corresponding to class 4 of cardiovascular failure in the Sequential Organ Failure Assessment (SOFA) score; a Simplified Acute Physiologic Score II of 25 or more; and written informed consent from the patients themselves or their representatives.

Patients were excluded if they were pregnant, if they had end-phase chronic disease, if they had a medical staff decision of limitation of care, if they had preliminary circulatory failure, if they had shock due to a urinary infection without bacteraemia, if they had peritonitis related to peritoneal dialysis or trauma, or if they were participating in another clinical trial.

### Treatments

Patients were randomly assigned in a 1:1 manner to receive either sodium selenite or matching placebo for 10 days. Treatments (Laboratoires Aguettant, Lyon, France) were conditioned in ampoules containing 1 mg selenium as sodium selenite diluted in 48 ml saline and were administered intravenously by continuous infusion (2 ml/hour) at the following doses, expressed in selenium content: 4,000 μg on the first day and 1,000 μg/day on the nine following days. Randomization was stratified on each centre by blocks of four. In each centre, sequentially identical numbered boxes containing the whole treatment for each patient were delivered to the investigator by the pharmacist following the order of the randomization list. All patients, medical and nursing staff, and pharmacists remained blinded throughout the study period.

### Data collection at inclusion

#### Clinical variables

The following data were recorded at inclusion. First, the baseline characteristics, the underlying condition assessed by the McCabe score, the length of hospital stay and the time in the ICU before enrolment were recorded. The severity of illness was also assessed by vital signs, the Simplified Acute Physiologic Score II and the SOFA score. Finally, interventions including the volume of fluid infusion during the previous 24 hours, the type and doses of vasopressors, and the mechanical ventilation conditions were recorded.

#### Laboratory variables

Haematological and biochemical analysis, arterial lactate and blood gases (allowing the determination of the PaO_2_/FiO_2 _ratio), blood cultures and cultures of specimen drawn from the site of infection were carried out systematically. Thyroid function was assessed by triiodothyronine, thyroxine, and thyroid stimulating hormone. The analytical methods used to assess all laboratory variables were the routine methods performed in each hospital. These methods are standardized according to French quality guidelines in medical biology.

### Follow-up

Patients were followed up for 1 year after randomization or until death, depending on which occurred first. The following variables were collected on days 2, 3, 4, 7, 10 and 14 after randomization: vital signs, SOFA score, standard laboratory tests, PaO_2_/FiO_2 _ratio, and interventions. Thyroid function was assessed on day 7 and on day 14. Cultures of specimens drawn from any new site of infection were performed throughout the ICU stay. The occurrence of nosocomial pneumonia and the need for dialysis was noted throughout the ICU stay. In addition, the patient's status at ICU discharge, at hospital discharge and 1 year after randomization was recorded.

### Efficacy endpoints

The primary endpoint was the time to vasopressor therapy withdrawal during the ICU stay. Secondary endpoints were the duration of mechanical ventilation, the ICU and hospital lengths of stay, and the mortality rates at ICU, at hospital discharge, and at 7, 14, 28 and 180 days and 1 year after randomization.

### Safety endpoints

The following adverse events that could potentially be related to selenium toxicity were closely monitored: refractory shock, cardiac insufficiency, acute respiratory distress syndrome, hepatitis cytolysis, epilepsy, polyradiculonevritis, bleeding or coagulation disorders, and worsening of organ failure [7,21-23,25].

All serious adverse events were reported by investigators and were transmitted to the regulatory authorities according to the International Conference on Harmonisation of Technical Requirements for Registration of Pharmaceuticals for Human Use (Revision of the ICH Guideline on Clinical Safety Data Management – Data Elements For Transmission of Individual Case Safety Reports, E2B(R3), current step 2, 12 May 2005). All serious adverse events were blindly analysed and the degree of suspected relatedness of selenium to event(s) was assessed.

### Sample size and statistical analysis

This phase II study arbitrarily planned to include 60 patients (30 in each group) in order to assess the opportunity of a larger phase III trial. Making the hypothesis that the percentage of patients free of catecholamine at 10 days (end of study treatment) would be 60%, the sample size would have allowed the detection of an absolute increase of 25% of this percentage of patients in a two-sided test performed with a type I error of 5% and a power of 80%.

Statistical analysis was performed with SAS statistical software (V9.1; SAS Institute, Cary, NC, USA). Data are presented as the mean ± standard deviation for continuous variables unless otherwise noted, and as numbers with corresponding percentages for qualitative variables. Comparisons between groups were performed using the Student *t *test or Wilcoxon rank sum test as appropriate for continuous variables, and using the chi-square test, the Fisher exact test or the Cochrane–Mantel–Haenzel test as appropriate for categorical variables. Cumulative event curves were constructed by the Kaplan–Meier method and the effect of treatment was analysed using the log-rank test. All analyses were performed according to the intent-to-treat principle (all randomized patients were analysed according to the treatment group in which they were assigned). All reported *P *values are two sided, and *P *< 0.05 was considered significant.

## Results

**Figure 1:**
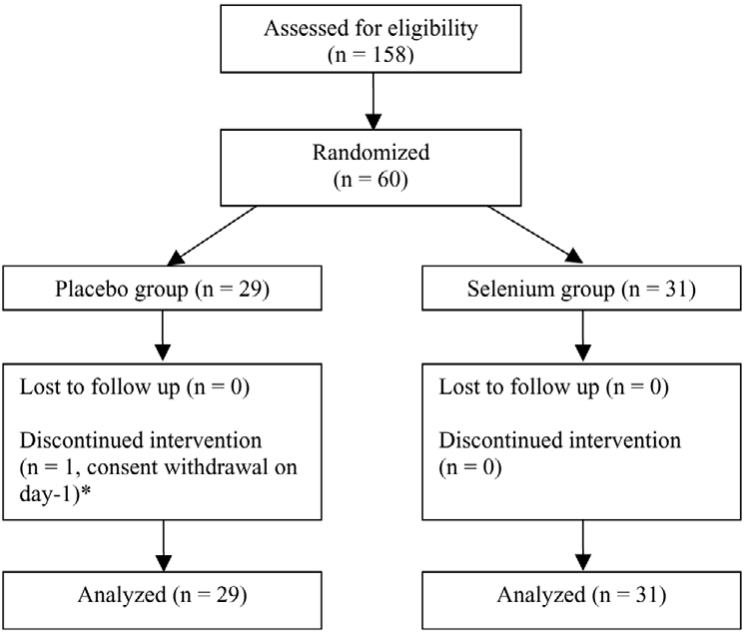
The participant flow diagram. *Use of data was permitted by the patient.

Between 8 February 2002 and 12 March 2004, a total of 60 patients were randomized (29 in the placebo group and 31 in the selenium group). All patients were followed up for the entire study period and were analysed as shown in Figure 1 (an intention-to-treat analysis).

### Characteristics of patients at inclusion

There was no significant difference between the two patient groups for the general characteristics at randomization except for the admission category, which showed a greater proportion of patients of medical origin in the selenium group (Table 1). The majority of the patients were included within 48 hours after ICU admission, with no significant difference between groups (79% and 90% in the placebo and selenium groups, respectively; *P *= 0.405).

**Table 1:** General characteristics at randomisation

Characteristic	Placebo group (*n *= 29)	Selenium group (*n *= 31)	*P *value
Age (years)	69 ± 12	66 ± 14	0.354
Male/female (*n*)	18/11	20/11	0.844
McCabe classification			
No disease	3 (10)	4 (13)	0.713
Nonfatal disease	16 (55)	14 (45)	
Ultimately fatal disease	10 (35)	13 (42)	
Rapidly fatal diseases	0 (0)	0 (0)	
Level of activity limitation^a^			
A	2 (7)	5 (16)	0.548
B	15 (52)	10 (32)	
C	7 (24)	6 (20)	
D	5 (17)	10 (32)	
Prior or pre-existing disease			
Hypertension	14 (48)	10 (32)	0.206
Coronary artery disease	6 (21)	2 (7)	0.140
Congestive heat failure	2 (7)	6 (19)	0.257
Chronic pulmonary disease	7 (24)	9 (29)	0.668
Diabetes	5 (17)	7 (23)	0.605
Liver disease	3 (10)	0 (0)	0.107
Cancer	4 (14)	7 (23)	0.379
Length of hospital stay before enrolment			
<14 days	24 (83)	29 (93)	0.064
14–28 days	4 (14)	0 (0)	
≥ 28 days	1 (3)	2 (7)	
Admission category			
Medical	18 (62)	29 (94)	0.003
Emergency surgery	10 (35)	2 (6)	
Elective surgery	1 (3)	0 (0)	

Data presented as the mean ± standard deviation for quantitative variables and *n *(%) for qualitative variables. ^a^Levels of activity limitation defined as follows: A, prior good health; B, mild to moderate limitation of activity because of chronic medical problem; C, chronic disease producing serious but not incapacitating restriction of activity; and D, severe restriction of activity due to disease.

The severity of illness at randomization was similar between the two groups except that the blood haemoglobin concentration was higher in the selenium group (Table 2). The origin of sepsis was mainly pulmonary, with a significantly higher rate of pneumonia in the selenium group, followed by peritoneal sepsis in the two groups (Table 3). In the selenium group, purely pulmonary infection was significantly twice more frequent and multisite infection was significantly four times less observed compared with the placebo group. The type of organism involved did not significantly differ between the two groups (Table 3).

**Table 2:** Severity of illness at randomisation

Variable	Placebo group (*n *= 29)	Selenium group (*n *= 31)	*P *value
Temperature (°C)	37.9 ± 1.3	37.9 ± 1.1	0.778
Mean arterial pressure (mmHg)	61 ± 22	64 ± 17	0.343
Heart rate (beats/min)	121 ± 38	121 ± 28	0.938
Simplified Acute Physiologic Score II at inclusion	62 ± 13	61 ± 20	0.824
Sequential Organ Failure Assessment score at inclusion	12 ± 3	11 ± 3	0.161
Haemoglobin (g/dl)	9.9 ± 2.0	11.3 ± 1.9	0.005
Leucocytes (x10^3^/μl)	14.0 ± 12.2	15.7 ± 9.1	0.243
Platelets (x10^3^/μl)	210 ± 163	253 ± 156	0.237
Arterial lactate (mmol/l)	4.2 ± 3.9	3.8 ± 2.4	0.600
PaO_2_/FiO_2 _(kPa)	21.7 ± 13.4	19.8 ± 14.7	0.329
Fluid infusion (l)^a^	3.3 ± 2.4	2.8 ± 1.9	0.436
Vasopressor therapy at randomization^b^			
Dopamine	21 (72)	19 (61)	0.361
Dobutamine	8 (28)	7 (23)	0.655
Epinephrine	12 (41)	14 (45)	0.768
Norepinephrine	19 (66)	13 (42)	0.067

Data presented as the mean ± standard deviation for quantitative variables and *n *(%) for qualitative variables. ^a^Amount of fluid infused during the previous 24 hours. ^b^Dopamine at any dose; dobutamine, epinephrine and norpinephrine at doses corresponding to the inclusion criteria.

**Table 3:** Origin of sepsis and type of organism involved

Characteristic	Placebo group (*n *= 29)	Selenium group (*n *= 31)
Site of infection		
Lung only	9 (31)	19 (61)^a^
Abdomen only	5 (17)	2 (6)
Urinary tract only	1 (3)	0 (0)
Other (one site only)^b^	3 (10)	7 (23)
More than one site	11 (38)	3 (10)^c^
At least one positive blood culture	12 (41)	8 (26)
Type of positive culture (any site)		
Purely Gram-positive	9 (31)	4 (13)
Purely Gram-negative	10 (34)	5 (16)
Anaerobes only	2 (7)	0 (0)
Mycobacterium	0 (0)	1 (3)
Mixed	7 (24)	11 (35)

Data presented as *n *(%). ^a^*P *= 0.019. ^b^Other sites included bone, catheter-related infection, septicaemia, central nervous system or oropharyngeal infections. ^c^*P *= 0.010.

### Efficacy

**Figure 2:**
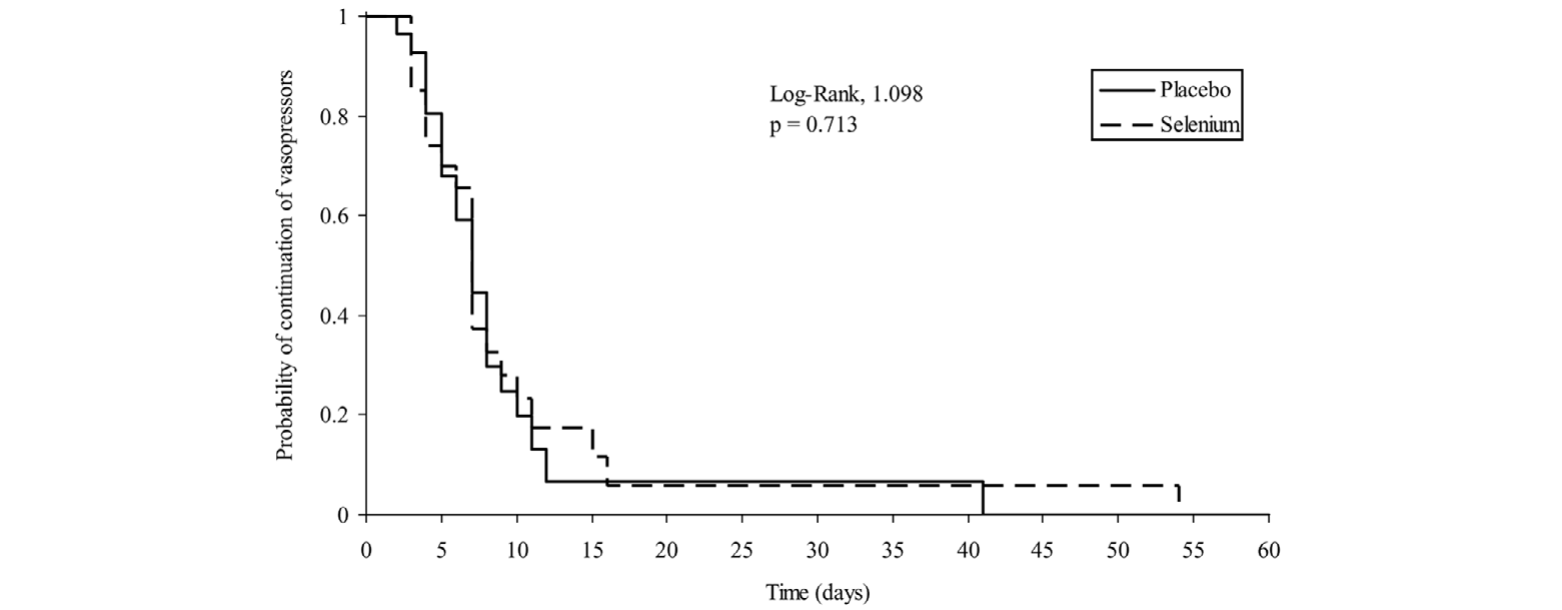
Time to vasopressor therapy withdrawal.

**Figure 3:**
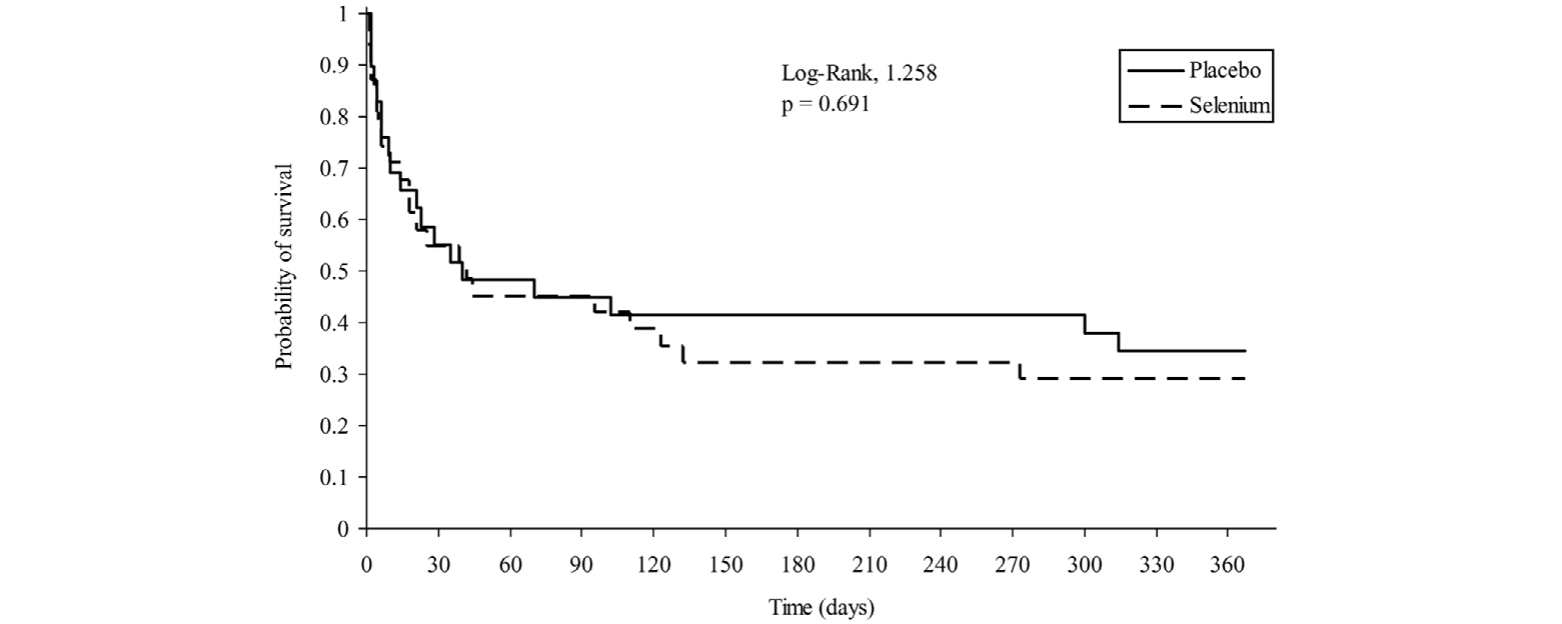
One year survival distribution. Mortality rates were 45% vs. 45% (p = 0.59), on day 28; 59% vs. 68% (p = 0.32) at 6 month; and 66% vs. 71% (p = 0.43) at one year in the placebo and selenium groups, respectively.

There was no significant difference between the two groups for time to vasopressor therapy withdrawal (Figure 2). The median time to vasopressor therapy withdrawal was 7 days in both groups (95% confidence interval = 5–8 and 6–9 in the placebo and selenium groups, respectively; log-rank, *P *= 0.713). The vasopressor-free rate on day 10 was 86% and 82% in the placebo and selenium groups, respectively (*P *= 0.775). The median (interquartile range) duration of mechanical ventilation was 14 (8–23) days in the placebo group and was 19 (7–34) days in the selenium group, respectively (*P *= 0.762). The median (interquartile range) ICU and hospital lengths of stay did not differ between the placebo and selenium groups (18 (10–31) days versus 21 (7–40) days, respectively, for the ICU length of stay; *P *= 0.836; and 33 (11–51) days versus 25 (7–68) days, respectively, for the hospital length of stay; *P *= 0.704). The mortality rates at ICU discharge, at hospital discharge, and at 7, 14, 28, and 180 days and 1 year after randomization were also similar in the two groups (Figure 3).

The SOFA score did not differ between groups during the 14 days of follow-up, and neither did the PaO_2_/FiO_2 _ratio and the triiodothyronine, thyroxine, and thyroid stimulating hormone levels (data not shown). The percentages of patients who had at least one nosocomial pneumonia event during the ICU stay were similar in the two groups (45% versus 55% for the placebo and selenium groups, respectively; *P *= 0.438), and the number of days free of dialysis were also similar in the two groups (26 ± 49 days versus 37 ± 55 days in the placebo and selenium groups, respectively; *P *= 0.303).

### Safety

At least one serious adverse event occurred in 62% and in 81% of the patients in the placebo and selenium groups, respectively (*P *= 0.111). The type of adverse event did not significantly differ between the two groups (Table 4), even though there was a trend to a higher rate of multiorgan failure in the selenium group (*P *= 0.09). None of these adverse events were classified as 'possibly' or 'probably' related to the study treatment.

**Table 4:** Incidence and type of serious adverse events

Variable	Placebo group (*n *= 29)	Selenium group (*n *= 31)	*P *value
At least one serious adverse event	18 (62)	25 (81)	0.111
Type of event			
Respiratory failure	1 (3)	5 (16)	0.196
Cardiac failure	1 (3)	1 (3)	1.000
Renal failure	1 (3)	0 (0)	0.483
Ischaemic event	6 (21)	2 (7)	0.134
Intracranial haemorrhage	1 (3)	1 (3)	1.000
Refractory shock	6 (21)	5 (16)	0.648
Multiorgan failure	4 (14)	10 (32)	0.091
Superinfection	2 (7)	1 (3)	0.606
Others^a^	0 (0)	2 (7)	0.492

Data presented as *n *(%). ^a^Includes one thrombocytopenia and one radial artery pseudoaneurysm.

## Discussion

In our study, the administration of 4,000 μg selenium, as a continuous infusion of sodium selenite, followed by 1,000 μg selenium per day during the nine following days was safe, but did not have any effect on the weaning of catecholamines. Moreover, there were no positive effects on the duration of mechanical ventilation, the ICU and hospital lengths of stay, and the mortality rates, as well as on the occurrence of nosocomial pneumonia and the need for renal replacement.

Differences between groups were found among few baseline characteristics due to a failure of randomization. For example, there was a higher rate of pneumonia in the treated group. The results of the study were far from significance, however, and it is probable that these imbalances do not impact the conclusions.

Our results do not agree with previous findings in sepsis trials. This is all the more surprising since we specifically included severe septic shock patients who were supposed to be the most responsive to selenium administration [35-37]. The fact that we did not observe any reduction of the hospital length of stay and infection rates, especially for nosocomial pneumonia, did not observe any effect on organ failure assessment, particularly renal failure, and observed no decrease in mortality also contrasts with the results of previous studies [35,36,38-43].

This absence of a beneficial effect of selenium treatment could theoretically be related to the small size of the study allowing conclusions only on the main endpoint. One must, however, underline that there was no trend to efficacy. Discrepancies could also be explained by differences in the type and severity of patients and/or in the therapeutic schedule. For example, mainly burn patients and trauma patients were included in the studies of Berger and colleagues instead of septic shock patients [38-41]. Selenium was administered as sodium selenite but daily doses were less than 500 μg, matched to substitute for losses, and they were administered in association with multi-antioxidant trace elements and sometimes with antioxidant multivitamins. The studies of Angstwurm and colleagues, of Kuklinski and colleagues, and of Zimmerman and colleagues, and the Selenium in Intensive Care studies, were respectively performed in patients with severe systemic inflammatory response syndrome, with acute pancreatitis or with sepsis syndrome instead of in patients with severe septic shock [35,36,42,43]. In these studies, doses ranged from 500 to 2,000 μg on the first day and the durations of intravenous administration were 9 days, 6 days, 28 days and 14 days, respectively, all with decreasing doses.

Another possible explanation for the absence of an effect could be an incipient toxicity of sodium selenite counterbalancing the moderate beneficial effect related to selenium infusion [44]. Indeed, it is well known in nutrition that trace element supplementation, particularly for selenium, is characterized by a dose–response curve with a plateau that is followed by toxicity if doses are increased [28,45,46]. These data, when considered together, may therefore suggest that the dose used in the present trial was beyond the optimal dose supporting immune defence.

It is important to note that, in the studies of Kuklinski and colleagues and of Zimmerman and colleagues, and in the Selenium in Intensive Care studies – which respectively showed 90%, 35% and 10% reductions of mortality rates – sodium selenite was administered using a bolus injection for the first administration. Moreover, one study used a similar scheme of continuous administration as the present study [47], in which administration was performed on 70 pancreatitis patients and the first dose of sodium selenite was 2 mg followed by 4 days at 300 μg/day. Their study failed to find any benefit, especially on mortality. To reduce the binding of NF-κB to DNA with selenite *in vivo*, therefore, a bolus administration is perhaps needed to reach high selenite blood concentrations that could not be attained by continuous administration [32,33,46]. Experimental animal studies are required to answer these questions.

We did not observe any of the predefined adverse events related to selenium as sodium selenite, despite using higher doses than those usually used in experimental studies (250–1,000 μg/day selenium) [8] that are far above previous recommendations (less than 700 μg/day selenium) [23,29-31]. Since a unique ingestion of 4 mg selenium is considered nontoxic in healthy man [22], we chose to administer sodium selenite corresponding to 4,000 μg selenium in septic shock patients using a continuous administration, rather than a bolus, to limit the risk of toxicity. This dose was followed by a 9-day administration of selenium as sodium selenite for antioxidant purposes. At these doses, the occurrence of side effects that could be related to the pro-oxidative properties of selenite were similar in the two groups, such as an increase in catecholamine requirement, cardiac insufficiency, acute respiratory distress syndrome, hepatitis cytolysis, epilepsy, polyradiculonevritis, and bleeding or coagulation disorders [7,21-23,25,44].

## Conclusion

In the present study, a continuous infusion of high doses of sodium selenite corresponding to 4 mg selenium the first day and 1 mg/day for the nine following days had no obvious toxicity but did not improve the clinical outcome in septic shock patients. The results of this nonpositive study may be related to the small sample size, to the inadequate dose and/or modalities of administration, or to an incipient toxicity of selenite counterbalancing a moderate beneficial effect. These results may paradoxically highlight interest in a selenite blood peak concentration at an early stage of sepsis. Considering that a dose above 500–800 μg/day selenium should not be administered in routine practice in ICU patients outside experimental situations, and considering the potential interest of bolus administration, septic shock animal studies are first needed to test the efficacy of this approach as well as the mechanism of its action.

## Key messages

• Sodium selenite is a pro-oxidant compound. Through secondary incorporation into selenoenzymes, however, the compound's selenium atom has antioxidant properties.

• Both the pro-oxidant and antioxidant properties may be sequentially useful in septic shock treatment.

• Continuous infusion of high doses of sodium selenite was not associated with detectable adverse effects but did not improve the clinical outcome in septic shock patients.

• Interest in an early peak of sodium selenite for septic shock treatment needs further investigation.

## Abbreviations

FiO_2 _= fraction of inspired oxygen; ICU = intensive care unit; NF = nuclear factor; PaO_2 _= arterial partial pressure of oxygen; SOFA = Sequential Organ Failure Assessment.

## Competing interests

XF is the co-inventor of patent FR 98 10889, PCT N°FR 99/02.66 (delivered: US 6,844,012 B1, Au 760 534; EP 1107767), and has ownership of the corresponding patent. XF is the sole shareholder of a small start-up named SÉRÉNITÉ-Forceville.

DV is the co-inventor of patent FR 98 10889, PCT N°FR 99/02.66 (delivered: US 6,844,012 B1, Au 760 534; EP 1107767).

The other authors (BL, DA, GB, J-MK, EC, HG, J-LS, AC and EB) declare that they have no competing interests.

## Authors' contributions

XF obtained the financing, developed the link with the administrative staff of Meaux Hospital – especially for pharmaceutical aspects – and the coordination between centres, participated in the study design and execution, in interpretation of the data, and in the writing of the manuscript. BL coordinated the monitoring of the study, performed the medical analysis of adverse events, and participated in the statistical analysis, in the interpretation of the data, and in the writing of the manuscript. DA, GB, J-MK, EC, HG, and J-LS participated in the execution of the study and in the interpretation of the data. DV participated in the study design and the interpretation of the data. AC participated in the study design and execution, and in the writing of the manuscript. EB realized the methodology of the study, coordinated the monitoring, data management, statistical analysis, interpretation of the data, and analysis of adverse events, and participated in the writing of the manuscript. All authors read and approved the final manuscript.

## Appendix: study organization

*Study chairmen*: Dr Xavier Forceville (Principal Investigator) and Prof. Eric Bellissant (Methodologist).

*Monitor and serious adverse events management*: Dr Bruno Laviolle, Centre d'Investigation Clinique INSERM 0203, Unité de Pharmacologie Clinique, Hôpital de Pontchaillou, Rennes.

*Monitoring*: Christelle Tual, Centre d'Investigation Clinique INSERM 0203, Unité de Pharmacologie Clinique, Hôpital de Pontchaillou, Rennes.

*Data management and statistical analysis*: Valérie Turmel, Centre d'Investigation Clinique INSERM 0203, Unité de Pharmacologie Clinique, Hôpital de Pontchaillou, Rennes.

*Quality Assurance*: Catherine Mouchel, Centre d'Investigation Clinique INSERM 0203, Unité de Pharmacologie Clinique, Hôpital de Pontchaillou, Rennes.

*Promotor*: Centre Hospitalier de Meaux, Meaux Cedex.

*Funding*: Grant from the Ministry of Health, France, PHRC 1998 (XF).

*Drug*: Unrestrictive grant from Aguettant, Lyon, France, for manufacturing study drugs that were distributed by SODIA, Reims, France.

*Labels*: This study received the labels of the Société de Réanimation de Langue Française and of the Société Francophone d'Etude et de Recherche *sur *les Eléments Toxiques et Essentiels.
